# Large tumor thrombus extending into the right atrium through the inferior vena cava due to hepatocellular carcinoma: A case report

**DOI:** 10.1016/j.radcr.2024.11.026

**Published:** 2024-12-06

**Authors:** Georgios Benetos, Angeliki Vakka, Eirini Solomou, Vasiliki Katsi, Konstantinos Tsioufis, Konstantinos Toutouzas

**Affiliations:** a First Department of Cardiology, Hippokration Hospital, National & Kapodistrian University of Athens, Greece; b Cardiac CT Department, Lefkos Stavros Clinic, Athens Greece; c 3rd Department of Internal Medicine, General Hospital of Nikaia, Piraeus, Greece; d Cardiology Department, Hippokration Hospital, Athens, Greece

**Keywords:** Tumor thrombus, Right atrium, Hepatocellular carcinoma, Case report

## Abstract

This is a case report of a 54-year-old patient with hepatocellular cancer under palliative chemotherapy who admitted with dyspnea on minimal exertion and peripheral oedema over the past 5 days. Echocardiogram revealed a large echogenic mass in the right atrial cavity which did not enhance with intravenous echo contrast agent, and a distended inferior vena cava (IVC) which was occluded by echogenic material with no signs of flow. To distinguish with accuracy if the thrombus was a bland or tumor thrombus, contrast-enhanced Computed Tomography (CT) was performed. CT Pulmonary Angiography and abdominal contrast-enhanced CT showed a distended and occluded IVC by a mass that extended to the right atrium and enhanced with intravenous contrast agent, and thus the mass was considered as a tumor thrombus. Due to the impaired performance status and liver function of the patient, supportive treatment was preferred instead of a surgical or radiological intervention. Large tumor thrombus extending into the right atrium through the inferior vena cava due to hepatocellular carcinoma has a rare incidence and is associated with a poor prognosis.

## Introduction

Hepatocellular carcinoma (HCC), which is a global health challenge with an increasing incidence, is the most common type of primary liver cancer and accounts for approximately 75–90% of primary liver cancers [1,2]. HCC tends to invade liver vasculature and thus is frequently complicated by portal venous tumor thrombosis (PVTT), which indicates advanced disease and is associated with poor prognosis according to the Barcelona Clinic Liver Cancer Classification (BCLC) [3]. Invasion of the tumor thrombus into the inferior vena cava (IVC) and the right atrium (RA) has a rare incidence of 0.67% to 4.1% according to autopsies of patients with HCC and is associated with a worse prognosis compared to PVTT [[4], [5]–6]. We report the rare case of a patient with HCC and a large tumor thrombus extending into the RA through the IVC.

## Case report

**Fig. 1:**
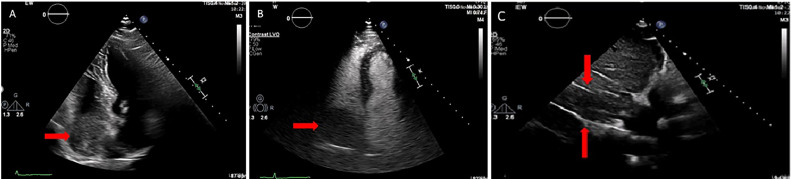
(A) Transthoracic echocardiography revealed a large echogenic mass in the right atrium (red arrow), (B) Contrast echocardiography showed that the mass in the Fig. 1A does not enhance with intravenous contrast agent, indicating that this mass may be a bland thrombus (red arrow), (C) Inferior vena cava appears distended and occluded with echogenic material and no signs of flow (between the red arrows) in the transthoracic echocardiogram.

**Fig. 2:**
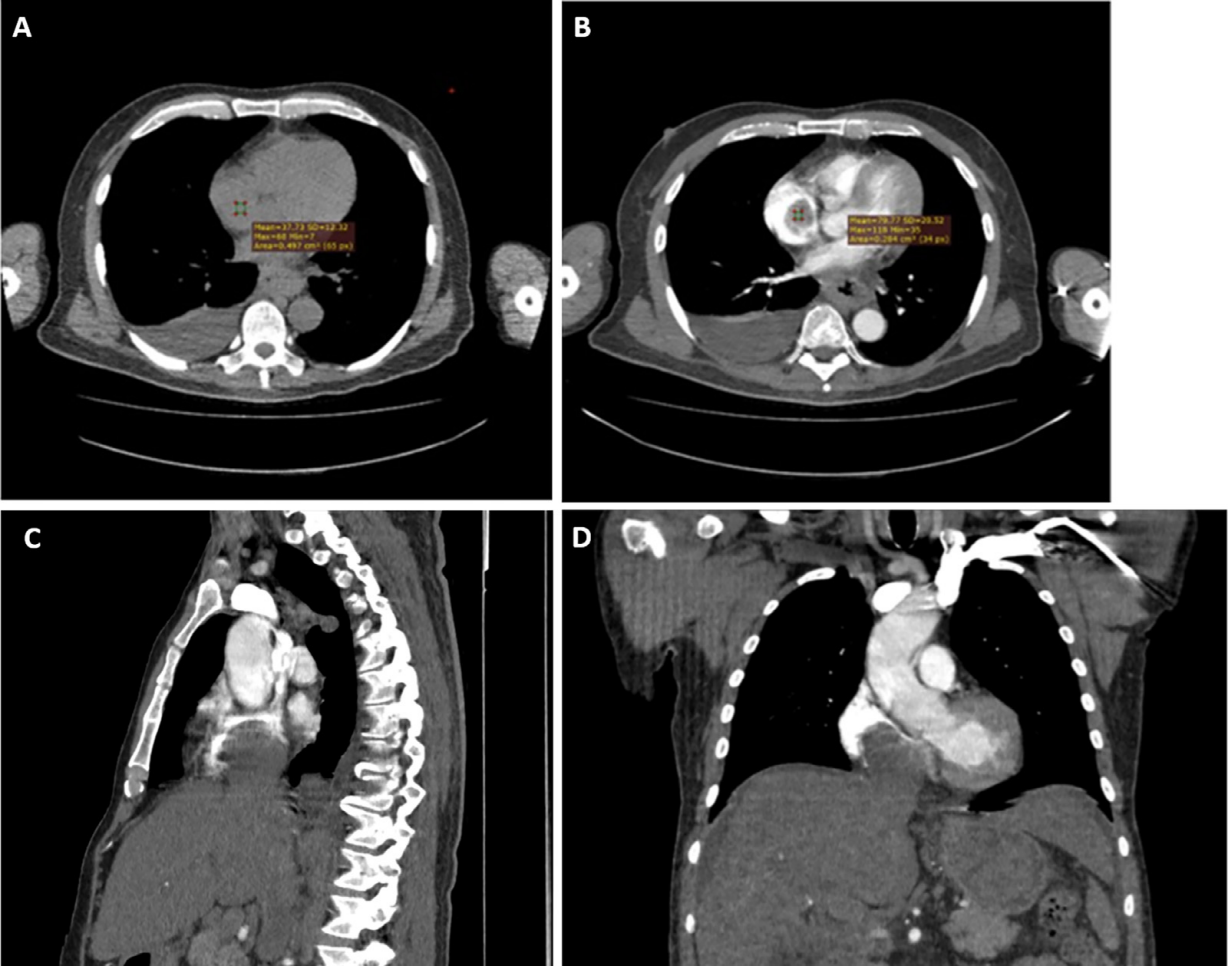
(A) Computed tomography pulmonary angiography before contrast enhancement showed that the mass extends from inferior vena cava to the right atrium, (B) Computed tomography pulmonary angiography after contrast enhancement revealed that this mass enhances with contrast agent, indicating that this mass may be a tumor thrombus, (C) Sagittal view showing the tumor thrombus extending from inferior vena cava to the right atrium, (D) Coronal view showing the tumor thrombus extending from inferior vena cava to the right atrium.

A 54-year-old patient with hepatocellular cancer under palliative chemotherapy presented to the emergency department with dyspnea on minimal exertion and peripheral oedema over the past 5 days. His medical history also included diabetes mellitus type 2, coronary artery disease, and cirrhosis due to chronic hepatitis C infection. On assessment he was hemodynamically unstable. Laboratory results showed alpha-fetoprotein (AFP) levels of 1490300 ng/dl and albumin-bilirubin (ALBI) score of -1.13, while the Child-Pugh grade was C. Echocardiogram revealed a large echogenic mass in the right atrial cavity (Fig. 1A, red arrow) which did not enhance with intravenous echo contrast agent (Fig. 1B, red arrow). Inferior vena cava (IVC) appeared distended and occluded by echogenic material with no signs of flow (Fig. 1C, between red arrows). The right and left ventricles were of normal size and function, and there were no valvular abnormalities. Low molecular weight heparin was initiated. Thoracic and abdominal contrast-enhanced Computed Tomography (CT) showed PVTT, as well as a distended and occluded IVC by a mass that extended to the right atrium (Fig. 2A, C, D) and enhanced with intravenous contrast agent (Fig. 2B, C, D), and thus the mass was considered as a tumor thrombus. CT Pulmonary Angiography (CTPA) was negative for pulmonary embolism.

Due to the performance status of the patient, the advanced stage of liver disease, his liver function and his chronic liver disease, supportive treatment was preferred instead of a surgical or radiological intervention. Imaging with Cardiac Magnetic Resonance (CMR) was scheduled to confirm that the type of thrombus was tumor thrombus. However, the patient experienced a massive upper gastrointestinal hemorrhage with hematemesis on the day of hospital admission. Emergent endoscopy revealed large esophageal varices treated with 5 clips. Patient developed hepatic encephalopathy during hospitalization and deceased 5 days later.

## Discussion

Primary liver cancer is the sixth most frequently diagnosed cancer and the fourth most common cause of cancer mortality worldwide [7]. HCC is the most common form of liver cancer [1]. Significant risk factors for HCC include HBV infection, HCV infection, alcohol-associated liver disease, metabolic dysfunction–associated steatotic liver disease, and aflatoxins [8].

Hepatocellular carcinoma (HCC) tends to invade intrahepatic vessels, leading to the formation of tumor thrombus. It is estimated that approximately 10% to 60% of patients with HCC have PVTT at the time of diagnosis [9], while invasion of the tumor thrombus into the IVC and the RA has a 0.67% to 4.1% incidence according to autopsies of patients with HCC [4–6]. The presence of tumor thrombus is associated with a poorer prognosis [10]. Here we present the case of a patient with a large tumor thrombus extending into the right atrium through the inferior vena cava due to hepatocellular carcinoma.

Formation of bland thrombus can also occur in patients with HCC, since HCC results in changes in blood flow, endothelial dysfunction and hypercoagulability [11]. Although differentiation of tumor thrombus from bland thrombus in the portal vein may be challenging, it is important, as it has prognostic and therapeutic implications [12]. According to the European Association for the Study of the Liver (EASL) Guidelines, portal vein thrombosis can be distinguished from PVTT with high accuracy by using contrast-enhanced imaging techniques. Specifically, presence of arterial phase hyper-enhancement and high signal intensity within the obstructed vessel on diffusion-weighted MRI with high b-values are two specific imaging findings for PVTT [12]. Other imaging features that may be present in PVTT are intrathrombus neovascularity, appearance of vessel expansion, direct invasion of the portal vein by HCC, and continuity of the thrombus with HCC [13]. These features may be also used for the differentiation of tumor thrombus from bland thrombus in the IVC and the RA. A study of 467 patients with HCC listed for liver transplantation showed that the presence of ≥3 of the following noninvasive criteria (the A-VENA criteria): AFP >1000 ng/dL; venous expansion; thrombus enhancement; neovascularity; and continuity with HCC characterized PVTT with 100% sensitivity and 93.6% specificity [13]. In the case of our patient, venous expansion, thrombus enhancement, and elevated AFP levels were present, which are suggestive of a tumor thrombus.

The BCLC classification is the most widely accepted model for prognosis prediction and treatment recommendation in HCC, as it integrates characteristics of the tumor and liver function, as well as performance status of the patient [3]. Patients with portal vein invasion (PVI) and/or extrahepatic spread with preserved liver function and a performance status of 1-2 are classified into the category of BCLC stage C (advanced stage) [3]. It has been reported that patients with type II (left and/or right main PVI) and type III PVI (main trunk invasion or beyond) have poorer median survival than patients with type I PVI (segmental or sectoral PVI) [14].

According to the BCLC proposal, patients with BCLC stage C HCC should be evaluated for systemic therapy [3]. Retrospective studies also showed that transarterial chemoembolization (TACE) plus systemic therapy, and locoregional radiation therapy plus systemic therapy are associated with longer survival in patients with BCLC stage C HCC compared to those treated with systemic therapy alone [15,16]. TACE plus systemic therapy has been also used in HCC patients with IVC and RA tumor thrombosis [17]. However, our patient belonged to the BCLC stage D due to a performance status >2 and impaired liver function without the option of liver transplantation, and in this way palliative care was preferred.

## Conclusion

HCC with a tumor thrombus extending into the IVC and RA has a rare incidence and is associated with poor prognosis. Thrombus in the right atrium can be detected with echocardiography, however contrast-enhanced imaging techniques are needed to distinguish tumor thrombus from bland thrombus with accuracy.

## Patient consent

We confirm that written informed consent has been obtained from the involved patient or if appropriate from the parent, guardian, power of attorney of the involved patient(s); and, they have given approval for this information to be published in this case report (series).
